# Poly[diaqua­bis(μ-4-carb­oxy-2-propyl-1*H*-imidazole-5-carboxyl­ato-κ^3^ *N* ^3^,*O* ^4^:*O* ^5^)calcium(II)]

**DOI:** 10.1107/S1600536809052799

**Published:** 2009-12-12

**Authors:** Wen-Dong Song, Jian-Bin Yan, Shi-Jie Li, Dong-Liang Miao, Xiao-Fei Li

**Affiliations:** a College of Science, Guang Dong Ocean University, Zhanjiang 524088, People’s Republic of China

## Abstract

In the title complex, [Ca(C_8_H_9_N_2_O_4_)_2_(H_2_O)_2_]_*n*_, the Ca^II^ atom is eight-coordinated in a distorted square-anti­prismatic environment. The water-coordinated Ca atom is *N*,*O*-chelated by the monocarboxyl­ate anion; the carboxyl –CO_2_ portion engaged in chelation bears an acid hydrogen. The free –CO_2_ portion engages in bonding to adjacent Ca atoms. The Ca^II^ centres are connected through the ligand, forming a layer structure; the layers are linked by hydrogen bonds into a three-dimensional network.

## Related literature

For the potential uses and diverse structrual types of structures containing metals and *N*-heterocyclic carboxylic acids, see: Liang *et al.* (2002 ▶); Net *et al.* (1989 ▶); Nie *et al.* (2007 ▶).
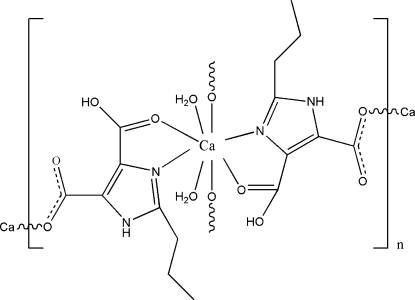


## Experimental

### 

#### Crystal data

[Ca(C_8_H_9_N_2_O_4_)_2_(H_2_O)_2_]
*M* *_r_* = 470.46
Monoclinic, 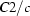

*a* = 12.703 (3) Å
*b* = 13.006 (3) Å
*c* = 11.697 (2) Å
β = 97.864 (2)°
*V* = 1914.3 (7) Å^3^
*Z* = 4
Mo *K*α radiation
μ = 0.40 mm^−1^
*T* = 273 K
0.32 × 0.24 × 0.20 mm

#### Data collection

Bruker APEXII area-detector diffractometer
Absorption correction: multi-scan (*SADABS*; Bruker, 2004 ▶) *T*_min_ = 0.884, *T*_max_ = 0.925
4830 measured reflections
1718 independent reflections
1504 reflections with *I* > 2σ(*I*)
*R*_int_ = 0.040

#### Refinement

*R*[*F* ^2^ > 2σ(*F* ^2^)] = 0.034
*wR*(*F* ^2^) = 0.099
*S* = 1.02
1718 reflections
144 parameters
3 restraints
H-atom parameters constrained
Δρ_max_ = 0.29 e Å^−3^
Δρ_min_ = −0.23 e Å^−3^

### 

Data collection: *APEX2* (Bruker, 2004 ▶); cell refinement: *SAINT* (Bruker, 2004 ▶); data reduction: *SAINT*; program(s) used to solve structure: *SHELXS97* (Sheldrick, 2008 ▶); program(s) used to refine structure: *SHELXL97* (Sheldrick, 2008 ▶); molecular graphics: *SHELXTL* (Sheldrick, 2008 ▶); software used to prepare material for publication: *SHELXTL*.

## Supplementary Material

Crystal structure: contains datablocks I, global. DOI: 10.1107/S1600536809052799/ng2702sup1.cif

Structure factors: contains datablocks I. DOI: 10.1107/S1600536809052799/ng2702Isup2.hkl

Additional supplementary materials:  crystallographic information; 3D view; checkCIF report

## Figures and Tables

**Table 1:** Hydrogen-bond geometry (Å, °)

*D*—H⋯*A*	*D*—H	H⋯*A*	*D*⋯*A*	*D*—H⋯*A*
N2—H2⋯O1^i^	0.86	2.01	2.859 (2)	171
O1*W*—H2*W*⋯O1^ii^	0.83	2.31	3.088 (2)	156
O1*W*—H1*W*⋯O3^iii^	0.84	2.12	2.947 (2)	172

Symmetry codes: (i) 
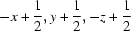
; (ii) 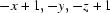; (iii) 
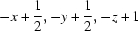
.

**Fig. 1.**
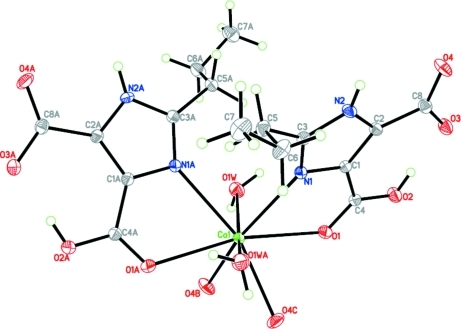
The structure of the title compound, showing the atomic numbering scheme. Non-H atoms are shown with 30% probability displacement ellipsoids(H atoms are represented by arbitrary spheres). [Symmetry codes:[(A)1 - x,y,1.5 - z;(B)-1/2 + x,1/2 + y,z;(C)1.5 - x,1/2 + y,1.5 - z.]

**Fig. 2.**
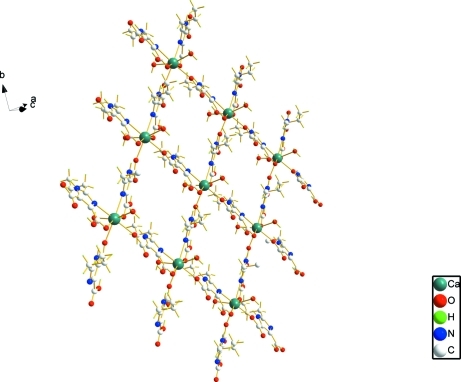
A view of an extended two-dimensional layer structure of the title compound.

**Fig. 3.**
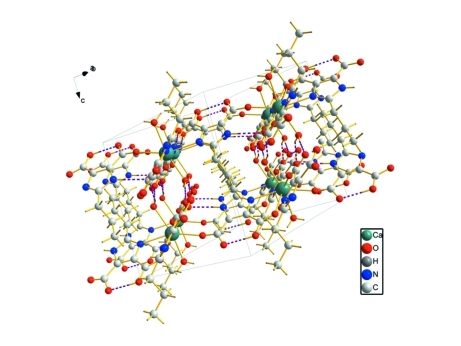
View the three-dimensional network.

## supplementary crystallographic information

### Comment

Recently, structures containing metals and N-heterocyclic carboxylic acids has attracted much attention, they can function as a multidentate ligand, exhibiting diverse structrual type and can be potentially used as functional materials (Nie *et al.*, 2007; Liang *et al.*,2002; Net *et al.*, 1989). In this paper, we report the synthesis and structure of a new Ca(II) complex obtained from 2-Propyl-1*H*- imidazole-4,5-dicarboxy with metal salts under hydrothermal conditions.

As illustrated in figure 1, the title complex molecule is eight-coordinated by two chelating rings [Ca—N=2.5998 (15)Å and Ca—O=2.605 (5) Å] and two carboxylate O atoms from two different 2-Propyl-1*H*-imidazole-4,5-dicarboxylate ligands and two water molecules, exhibiting a distorted square antiprismatic structure, the title Complex displays an extended two-dimensional layer structure constructed of quasi-squares, with four Ca atoms at the corners and 2-Propyl-1*H*-imidazole-4,5-dicarboxylate anions at each edge as linkers connecting two Ca atoms. the edge lengthes are equal, with a value of 9.0901 (16) Å. the angles of the rhombus are 88.650 (2)° and 91.350 (5)°(Fig. 2). Two dimensional layers are further linked by hydrogen bonds(Table 1), forming a three-dimensional network(Fig. 3)

### Experimental

A mixture of Ca(II)chloride (0.5 mmol, 0.055 g) and 2-propyl-1*H*-imidazole-4,5-dicarboxylic acid(0.5 mmol, 0.99 g) in 10 ml of distilled water was sealed in an autoclave equipped with a Teflon liner (20 ml) and then heated at 433k for 3 days. Crystals of the title compound were obtained by slow evaporation of the solvent at room temperature.

### Refinement

Carbon and nitrogen bound H atoms were placed at calculated positions and were treated as riding on the parent C or N atoms with C—H = 0.93 Å, N—H = 0.86 Å, and with *U*_iso_(H) = 1.2 *U*_eq_(C, N). The water H-atoms were located in a difference map, and were refined with a distance restraint of O—H = 0.84 Å; their *U*_iso_ values were refined.

### Crystal data

**Table:**

[Ca(C_8_H_9_N_2_O_4_)_2_(H_2_O)_2_]	*F*(000) = 984
*M**_r_* = 470.46	*D*_x_ = 1.632 Mg m^−^^3^
Monoclinic, *C*2/*c*	Mo *K*α radiation, λ = 0.71073 Å
Hall symbol: -C 2yc	Cell parameters from 3600 reflections
*a* = 12.703 (3) Å	θ = 1.4–28°
*b* = 13.006 (3) Å	µ = 0.40 mm^−^^1^
*c* = 11.697 (2) Å	*T* = 273 K
β = 97.864 (2)°	Block, white
*V* = 1914.3 (7) Å^3^	0.32 × 0.24 × 0.20 mm
*Z* = 4	

### Data collection

**Table:**

Bruker APEXII area-detector diffractometer	1718 independent reflections
Radiation source: fine-focus sealed tube	1504 reflections with *I* > 2σ(*I*)
graphite	*R*_int_ = 0.040
φ and ω scan	θ_max_ = 25.2°, θ_min_ = 2.3°
Absorption correction: multi-scan (*SADABS*; Bruker, 2004)	*h* = −13→15
*T*_min_ = 0.884, *T*_max_ = 0.925	*k* = −14→15
4830 measured reflections	*l* = −14→13

### Refinement

**Table:**

Refinement on *F*^2^	Secondary atom site location: difference Fourier map
Least-squares matrix: full	Hydrogen site location: inferred from neighbouring sites
*R*[*F*^2^ > 2σ(*F*^2^)] = 0.034	H-atom parameters constrained
*wR*(*F*^2^) = 0.099	*w* = 1/[σ^2^(*F*_o_^2^) + (0.0551*P*)^2^ + 1.470*P*] where *P* = (*F*_o_^2^ + 2*F*_c_^2^)/3
*S* = 1.01	(Δ/σ)_max_ < 0.001
1718 reflections	Δρ_max_ = 0.29 e Å^−^^3^
144 parameters	Δρ_min_ = −0.23 e Å^−^^3^
3 restraints	Extinction correction: *SHELXL97* (Sheldrick, 2008), Fc^*^=kFc[1+0.001xFc^2^λ^3^/sin(2θ)]^-1/4^
Primary atom site location: structure-invariant direct methods	Extinction coefficient: 0.0076 (9)

### Special details

**Table:**

Geometry. All e.s.d.'s (except the e.s.d. in the dihedral angle between two l.s. planes) are estimated using the full covariance matrix. The cell e.s.d.'s are taken into account individually in the estimation of e.s.d.'s in distances, angles and torsion angles; correlations between e.s.d.'s in cell parameters are only used when they are defined by crystal symmetry. An approximate (isotropic) treatment of cell e.s.d.'s is used for estimating e.s.d.'s involving l.s. planes.
Refinement. Refinement of *F*^2^ against ALL reflections. The weighted *R*-factor *wR* and goodness of fit *S* are based on *F*^2^, conventional *R*-factors *R* are based on *F*, with *F* set to zero for negative *F*^2^. The threshold expression of *F*^2^ > σ(*F*^2^) is used only for calculating *R*-factors(gt) *etc*. and is not relevant to the choice of reflections for refinement. *R*-factors based on *F*^2^ are statistically about twice as large as those based on *F*, and *R*- factors based on ALL data will be even larger.

### Fractional atomic coordinates and isotropic or equivalent isotropic displacement parameters (Å^2^)

**Table:**

	*x*	*y*	*z*	*U*_iso_*/*U*_eq_	
Ca1	0.5000	0.07420 (4)	0.2500	0.0189 (2)	
O1	0.33875 (11)	0.05035 (10)	0.36313 (12)	0.0262 (3)	
O1W	0.55495 (11)	0.10622 (11)	0.45862 (12)	0.0319 (4)	
H1W	0.5121	0.1347	0.4970	0.048*	
H2W	0.5769	0.0519	0.4904	0.048*	
O2	0.20033 (11)	0.11430 (10)	0.43530 (13)	0.0296 (4)	
H1	0.1663	0.1680	0.4316	0.044*	
O3	0.09452 (11)	0.27478 (11)	0.42169 (12)	0.0308 (4)	
O4	0.09469 (12)	0.42512 (10)	0.33195 (13)	0.0352 (4)	
N1	0.36595 (12)	0.22589 (11)	0.23987 (13)	0.0201 (4)	
N2	0.26433 (12)	0.36382 (11)	0.21921 (13)	0.0225 (4)	
H2	0.2403	0.4229	0.1951	0.027*	
C1	0.28670 (14)	0.21777 (14)	0.30920 (15)	0.0192 (4)	
C2	0.22299 (14)	0.30376 (14)	0.29747 (16)	0.0209 (4)	
C3	0.34950 (14)	0.31557 (14)	0.18572 (16)	0.0215 (4)	
C4	0.27622 (14)	0.12212 (14)	0.37333 (16)	0.0204 (4)	
C5	0.40949 (15)	0.36092 (15)	0.09774 (17)	0.0255 (4)	
H5A	0.4753	0.3231	0.0974	0.031*	
H5B	0.4277	0.4315	0.1189	0.031*	
C6	0.34718 (17)	0.35929 (17)	−0.02384 (18)	0.0334 (5)	
H6A	0.3399	0.2888	−0.0508	0.040*	
H6B	0.2764	0.3865	−0.0212	0.040*	
C7	0.40144 (19)	0.42208 (18)	−0.1082 (2)	0.0382 (6)	
H7A	0.4067	0.4924	−0.0832	0.057*	
H7B	0.3606	0.4184	−0.1835	0.057*	
H7C	0.4714	0.3952	−0.1113	0.057*	
C8	0.13058 (15)	0.33836 (15)	0.35251 (16)	0.0240 (4)	

### Atomic displacement parameters (Å^2^)

**Table:**

	*U*^11^	*U*^22^	*U*^33^	*U*^12^	*U*^13^	*U*^23^
Ca1	0.0189 (3)	0.0157 (3)	0.0229 (3)	0.000	0.0052 (2)	0.000
O1	0.0267 (7)	0.0187 (7)	0.0336 (8)	0.0026 (6)	0.0051 (6)	0.0014 (6)
O1W	0.0345 (8)	0.0327 (8)	0.0299 (8)	0.0035 (6)	0.0095 (6)	0.0011 (6)
O2	0.0303 (8)	0.0228 (7)	0.0384 (8)	0.0041 (6)	0.0140 (7)	0.0073 (6)
O3	0.0295 (8)	0.0300 (8)	0.0360 (8)	0.0045 (6)	0.0155 (6)	0.0036 (6)
O4	0.0385 (9)	0.0295 (8)	0.0394 (9)	0.0182 (6)	0.0119 (7)	0.0052 (6)
N1	0.0188 (8)	0.0190 (8)	0.0231 (8)	0.0000 (6)	0.0049 (6)	0.0001 (6)
N2	0.0250 (8)	0.0157 (8)	0.0275 (9)	0.0045 (6)	0.0059 (7)	0.0042 (6)
C1	0.0185 (9)	0.0192 (9)	0.0198 (9)	−0.0003 (7)	0.0023 (7)	−0.0013 (7)
C2	0.0219 (9)	0.0200 (9)	0.0212 (9)	0.0015 (7)	0.0046 (8)	−0.0011 (7)
C3	0.0207 (9)	0.0201 (9)	0.0236 (10)	−0.0007 (7)	0.0027 (8)	−0.0004 (7)
C4	0.0203 (9)	0.0191 (9)	0.0215 (10)	−0.0007 (7)	0.0020 (7)	−0.0016 (7)
C5	0.0230 (10)	0.0255 (10)	0.0288 (11)	−0.0018 (8)	0.0062 (8)	0.0047 (8)
C6	0.0311 (11)	0.0350 (12)	0.0332 (12)	−0.0093 (9)	0.0012 (9)	0.0059 (9)
C7	0.0419 (13)	0.0429 (13)	0.0294 (12)	−0.0077 (10)	0.0037 (10)	0.0069 (9)
C8	0.0235 (10)	0.0266 (10)	0.0219 (10)	0.0022 (8)	0.0035 (8)	−0.0017 (8)

### Geometric parameters (Å, °)

**Table:**

Ca1—O4^i^	2.4104 (14)	N1—C1	1.380 (2)
Ca1—O4^ii^	2.4104 (14)	N2—C3	1.354 (2)
Ca1—O1W	2.4798 (15)	N2—C2	1.362 (2)
Ca1—O1W^iii^	2.4799 (15)	N2—H2	0.8600
Ca1—N1^iii^	2.5982 (15)	C1—C2	1.376 (3)
Ca1—N1	2.5982 (15)	C1—C4	1.468 (3)
Ca1—O1	2.6048 (14)	C2—C8	1.484 (3)
Ca1—O1^iii^	2.6049 (14)	C3—C5	1.484 (3)
O1—C4	1.242 (2)	C5—C6	1.530 (3)
O1W—H1W	0.8378	C5—H5A	0.9700
O1W—H2W	0.8287	C5—H5B	0.9700
O2—C4	1.287 (2)	C6—C7	1.517 (3)
O2—H1	0.8200	C6—H6A	0.9700
O3—C8	1.284 (2)	C6—H6B	0.9700
O4—C8	1.228 (2)	C7—H7A	0.9600
O4—Ca1^iv^	2.4102 (14)	C7—H7B	0.9600
N1—C3	1.330 (2)	C7—H7C	0.9600
			
O4^i^—Ca1—O4^ii^	72.89 (8)	C3—N2—C2	108.98 (15)
O4^i^—Ca1—O1W	125.76 (5)	C3—N2—H2	125.5
O4^ii^—Ca1—O1W	71.69 (5)	C2—N2—H2	125.5
O4^i^—Ca1—O1W^iii^	71.69 (5)	C2—C1—N1	110.21 (16)
O4^ii^—Ca1—O1W^iii^	125.76 (5)	C2—C1—C4	130.27 (17)
O1W—Ca1—O1W^iii^	160.66 (7)	N1—C1—C4	119.30 (15)
O4^i^—Ca1—N1^iii^	156.52 (5)	N2—C2—C1	104.93 (16)
O4^ii^—Ca1—N1^iii^	107.72 (5)	N2—C2—C8	121.21 (16)
O1W—Ca1—N1^iii^	74.52 (5)	C1—C2—C8	133.83 (17)
O1W^iii^—Ca1—N1^iii^	90.68 (5)	N1—C3—N2	110.39 (16)
O4^i^—Ca1—N1	107.72 (5)	N1—C3—C5	128.07 (17)
O4^ii^—Ca1—N1	156.53 (5)	N2—C3—C5	121.51 (17)
O1W—Ca1—N1	90.68 (5)	O1—C4—O2	122.20 (17)
O1W^iii^—Ca1—N1	74.52 (5)	O1—C4—C1	118.94 (16)
N1^iii^—Ca1—N1	81.19 (7)	O2—C4—C1	118.82 (16)
O4^i^—Ca1—O1	73.86 (5)	C3—C5—C6	112.93 (16)
O4^ii^—Ca1—O1	94.96 (5)	C3—C5—H5A	109.0
O1W—Ca1—O1	69.83 (5)	C6—C5—H5A	109.0
O1W^iii^—Ca1—O1	112.64 (5)	C3—C5—H5B	109.0
N1^iii^—Ca1—O1	128.56 (5)	C6—C5—H5B	109.0
N1—Ca1—O1	63.74 (4)	H5A—C5—H5B	107.8
O4^i^—Ca1—O1^iii^	94.96 (5)	C7—C6—C5	111.95 (17)
O4^ii^—Ca1—O1^iii^	73.85 (5)	C7—C6—H6A	109.2
O1W—Ca1—O1^iii^	112.64 (5)	C5—C6—H6A	109.2
O1W^iii^—Ca1—O1^iii^	69.82 (5)	C7—C6—H6B	109.2
N1^iii^—Ca1—O1^iii^	63.74 (4)	C5—C6—H6B	109.2
N1—Ca1—O1^iii^	128.56 (5)	H6A—C6—H6B	107.9
O1—Ca1—O1^iii^	166.32 (6)	C6—C7—H7A	109.5
C4—O1—Ca1	121.13 (12)	C6—C7—H7B	109.5
Ca1—O1W—H1W	119.1	H7A—C7—H7B	109.5
Ca1—O1W—H2W	109.3	C6—C7—H7C	109.5
H1W—O1W—H2W	109.8	H7A—C7—H7C	109.5
C4—O2—H1	109.5	H7B—C7—H7C	109.5
C8—O4—Ca1^iv^	165.57 (15)	O4—C8—O3	124.05 (18)
C3—N1—C1	105.49 (14)	O4—C8—C2	119.22 (17)
C3—N1—Ca1	138.32 (12)	O3—C8—C2	116.72 (17)
C1—N1—Ca1	116.18 (11)		

Symmetry codes: (i) −*x*+1/2, *y*−1/2, −*z*+1/2; (ii) *x*+1/2, *y*−1/2, *z*; (iii) −*x*+1, *y*, −*z*+1/2; (iv) *x*−1/2, *y*+1/2, *z*.

### Hydrogen-bond geometry (Å, °)

**Table:**

*D*—H···*A*	*D*—H	H···*A*	*D*···*A*	*D*—H···*A*
N2—H2···O1^v^	0.86	2.01	2.859 (2)	171
O1W—H2W···O1^vi^	0.83	2.31	3.088 (2)	156
O1W—H1W···O3^vii^	0.84	2.12	2.947 (2)	172

Symmetry codes: (v) −*x*+1/2, *y*+1/2, −*z*+1/2; (vi) −*x*+1, −*y*, −*z*+1; (vii) −*x*+1/2, −*y*+1/2, −*z*+1.
